# Reduced Awareness for Osteoporosis in Distal Radius Fracture Patients Compared to Patients with Proximal Femur Fractures

**DOI:** 10.3390/jcm10040848

**Published:** 2021-02-19

**Authors:** Alexander Martin Keppler, Moritz Kraus, Matthias Blaschke, Nicole Thomasser, Christian Kammerlander, Wolfgang Böcker, Carl Neuerburg, Ulla Cordula Stumpf

**Affiliations:** 1 Department of General, Trauma and Reconstructive Surgery, University Hospital, Ludwig-Maximilians-University Munich, 81377 Munich, Germany; alexander.keppler@med.uni-muenchen.de (A.M.K.); moritz.kraus@med.uni-muenchen.de (M.K.); nicole.thomasser@uk-augsburg.de (N.T.); christian.kammerlander@med.uni-muenchen.de (C.K.); wolfgang.boecker@med.uni-muenchen.de (W.B.); ulla.stumpf@med.uni-muenchen.de (U.C.S.); 2 Krankenhaus der Barmherzigen Brüder München, Akademisches Lehrkrankenhaus der Technischen Universität München, Romanstraße 93, 80639 Munich, Germany; matthias.blaschke@barmherzige-muenchen.de; 3 Department of Gastroenterology, University Hospital Augsburg, 86156 Augsburg, Germany

**Keywords:** awareness, osteoporosis, proximal femur fracture, wrist fracture, distal forearm fracture

## Abstract

Purpose: The present study is aiming to evaluate patients’ awareness to participate in further diagnostics for osteoporosis and to find out if there are significant differences with regards to fracture site. Methods: Patients at risk for underlying osteoporosis (female >60 and male >70 years) undergoing surgical treatment for a distal radius fracture (DRF) or a proximal femur fracture (PFF) were asked to complete a questionnaire assessing the awareness for underlying osteoporosis. Furthermore, dual-X-ray absorptiometry (DXA) scans were analyzed. Results: Overall, 150 patients (w = 122/m = 28, mean age 79.9 years (±8.6)) were included, of these, 36 patients suffered a DRF and 114 patients a PFF. Of these, 68 out of the 150 patients (45.3%) considered that an examination was necessary, whereas in PFF patients the awareness was higher than in the DRF Group (41% vs. 32%). Conclusions: The patients’ willingness to undergo further diagnostics for osteoporosis was generally poor. DRFs are frequently accompanied by a lower limitation of quality of life compared to PFF, which might be causative for even poorer awareness in these patients. Especially younger patients (age 60–70 years) with a distal radius fracture seemed to underestimate osteoporosis.

## 1. Introduction

Elderly patients presenting with a distal radius (DRF) or proximal femur fracture (PFF) are at high risk of suffering from subsequent osteoporotic fractures due to reduced bone quality and accompanying risk of secondary falls [1].

Fractures of the distal radius (DRFs) are commonly one of the first fracture sites indicating underlying osteoporosis. In a trial conducted by Sakuma M. et al., investigating the average age at the time of an osteoporotic fracture, it has been shown that patients suffering a DRF are almost 20 years younger than patients suffering a hip-, vertebral- or proximal humerus fracture (average age at the time of DRFs was 60.2 years vs. average age of the before-mentioned fractures = 81.4; 77.7; 75.7 years, respectively) [2]. The authors concluded that early fracture prevention and continuous prevention strategies are of superior importance in osteoporotic patients. Thus, DRFs that occur after a fall from body height should be considered as an indicator for the presence of underlying osteoporosis, of which postmenopausal women and men at the age of >65 years are especially at risk [3]. Considering the latest epidemiologic data, the incidence of distal radius fractures is 278 per 100,000 patients, with an expected increase by 38% until 2050 [4]. Previous studies have shown that wrist fractures usually occur before proximal femur fractures, so if effective treatment can be initiated through early diagnosis, these fractures may be avoided, saving money as well as providing better care [5].

Proximal femur fractures are among the most common fractures in elderly patients and are expected to increase up to 6.3 million per year by 2050 due to demographic change [6]. In addition to a decline in daily activities and mobility, the mortality increased up to 20% in the first year [7,8]. Despite the high incidence of osteoporosis after PFF, the rate of adequate treatment is alarmingly low [9].

## 2. Materials and Methods

From May 2015 to December 2016, all patients (female >60 years or male >70 years) undergoing surgery due to a DRF or hip fracture in our department of trauma surgery at a maximum care university hospital were consecutively included in the investigation. In order to assess the patients’ awareness for osteoporosis, a questionnaire was prepared that included major risk factors for osteoporosis (according to the German Society of Osteology (Dachverband für Osteologie e.V.(DVO)) [10] as well as the general willingness of the patients to perform Osteoporosis Diagnostics and other screening programs offered by the health insurance companies (colonoscopy and mammography). Patients undergoing an outpatient treatment due to a DRF and those with the following conditions were excluded: organic brain disorders (i.e., delirium, dementia, etc.), language barrier and patients with additional fractures. The study was approved by the university ethics committee and registered under AZ 351-14.

### 2.1. Questionnaire

The questionnaire was specially developed to examine the awareness of osteoporosis, the willingness to diagnose osteoporosis and the risk factors according to DVO and Fracture Risk Assessment Tool Score (FRAX). The questionnaire contained information about the person, age, height and weight as well as current self-assessed health status by means of visual analogue and numerical rating scale.

### 2.2. Self-Assessment of Health Status by Rating Scale

Rating scales were used to assess a state with regard to a certain characteristic. The numerical rating scale in the questionnaire was a one-dimensional scale that enabled patients to describe their current state of health. According to World Health Organization (WHO) in 1947, health is a state of complete mental, physical and social well-being. Patients were asked to tick the level of the rating scale which corresponded to their subjective perception of the characteristic expression. Zero (0) corresponded to complete health (=completely healthy) and 10—severe illness (=very sick). In addition, a symbolic rating scale was used in the form of smileys to facilitate the use of older patients who no longer have the necessary abstraction for numerical rating scales. Furthermore, the physical health status of the patients was assessed using the ASA score (American Society of Anesthesiologists) obtained by colleagues from the department of anesthesiology prior to surgery.

ASA Classification was developed in 1941 by a committee of the American Society of Anesthetists as a score to estimate the patients’ operative risk based on their physical status [11]. The latest revision of the classification from 2014 contains six groups, of which only the first four were relevant to the classification of our patients. Group one included healthy patients who underwent elective operations. Group two included individuals with mild, well-controlled systemic diseases. Patients with more serious, non-life-threatening conditions were put into group three, and group four contained all individuals with serious disorders that pose a permanent threat to their lives. Group five are patients who are not expected to survive the following 24 h and Group six are brain-dead patients. These last two groups did not contribute to the current study [11].

In order to facilitate the answer and evaluation, additional items about the patient’s health, information and awareness for osteoporosis were generated in a dichotomous nominal scale as a “yes/no” response. Thus, information about known or diagnosed osteoporosis was collected, such as a persisting medication. In addition, according to their own subjective need for an osteoporosis examination and the self-assessed threat of the disease was raised. Participation in other screening examinations was assessed, such as mammography screening in females up to 70 years, regular prostate examinations in males and colonoscopy in both female and male patients over the last 10 years.

Risk factors for osteoporosis (according to the DVO Guideline 2014) [12] were collected to determine the individual 10-year-risk of fractures as well as the indication for basis and/or specific medication therapy of osteoporosis. Thus, the following aspects were included: Fractures that occurred after the age of 50 years (wrist, vertebral, thigh fractures), parental hip fractures, nicotine consumption, glucocorticoid intake, underlying diseases such as type 1 diabetes mellitus, rheumatoid arthritis, osteogenesis imperfecta, untreated hyperthyroidism, hypogonadism, chronic liver disease, malnutrition, alcohol consumption (≥1 bottle of beer/1 glass of wine daily), daily intake of dairy products, regular intake of vitamin D. For women additionally: menopause <45 years, regular intake of aromatase inhibitors.

### 2.3. Osteoporosis Screening

Also, the individual risk for osteoporotic fractures was assessed by assessment of physical activity, risk of falls, dietary habits and the individuals’ medication. Furthermore, basic osteoporosis laboratory tests, bone densitometry by dual-energy X-ray absorptiometry (DXA) and, if needed, X-rays of the spine, were carried out to identify prevalent vertebral body fractures according to our treatment algorithm adapted to our guidelines for osteoporosis [13].

## 3. Results

Overall, 150 patients (female = 122 (81%)/male = 28 (19%)) were included in the study at an average age of 79.9 years (±8.6), where 36 patients were treated for a DRF and 114 patients for a PFF. Patient characteristics including body mass index (BMI), age group distribution and ASA scores are shown in Table 1.

### 3.1. Questionnaire and Self-Assessment of Health Status by Rating Scale

On the basis of a numerical rating scale (0 = total health, 10 = severe illness), the study population at the time of the survey indicated an average score of 4.09 ± 2.50 as the subjective state of health.

On average, the patients in our total cohort had 1.85 ± 1.4 risk factors according to the DVO guidelines. The prevalence of the queried risk factors for osteoporosis is shown in Table 1. According to the Fracture Risk Assessment Tool Score (FRAX)-Score, “high risk” is associated with a 10-year fracture probability of ≥3% for hip and/or ≥20% for other large osteoporotic fractures, whereas “low risk” is considered for risk percentages below. Of the investigated patients, 61% (*n* = 91/150) had a 20% risk of a major osteoporotic fracture over the next 10 years. Up to 89% of the investigated patients were at risk to have another proximal femur fracture within the next ten years. No differences were found between DRF and proximal femur fracture patients.

Only 45% (*n* = 68) of the 150 patients were willing to undergo screening for underlying osteoporosis, which was lower than their willingness to undergo other screening programs (mammography at 63% (*n* = 77) and colonoscopy at 60% (*n* = 90)). No differences were found between DRF and hip fracture patients.

### 3.2. Testing for Osteoporosis and Bone Mineral Density

Overall, 114 out of 150 patients had not been diagnosed or screened for osteoporosis before their hospital stay. Eighty-three percent (*n* = 95) of the 114 patients were diagnosed to have underlying osteoporosis, 6% (*n* = 7) had osteopenia and 11% (*n* = 12) had a clinically evaluated high risk for osteoporosis. In these 12 patients, a later clarification in the outpatient osteoporosis clinic was recommended. 

Another 36 patients from the proximal femur group received no further DXA because osteoporosis was diagnosed according to the DVO guideline when certain aspects were present: low-energy trauma (fall from standing height, for example, after stumbling), X-rays show rarefied bone aspects, clinical signs of osteoporosis can be found and the age is for men >60 years and postmenopausal status for women [14].

**Figure 1:**
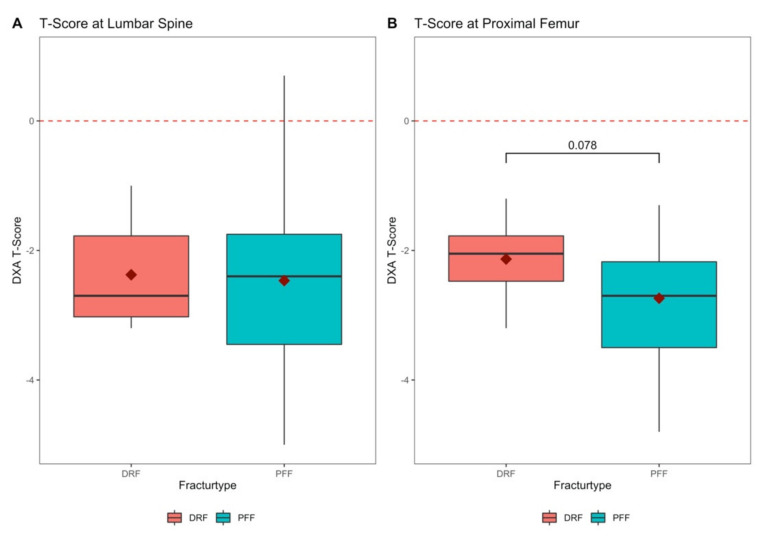
Bone mineral density of the lumbar spine and proximal femur. (**A**) Bone mineral density of the lumbar spine expressed in T-Score, with distal radius fractures (DRF) compared to patients with proximal femur fractures (PFF) (*p* = 0.815, U-test)); (**B**) Bone mineral density of the femoral neck expressed in T-score of patients with distal radius fractures (DRF) compared to patients with proximal femur fractures (PFF) (*p* = 0.078, U-test).

**Figure 2:**
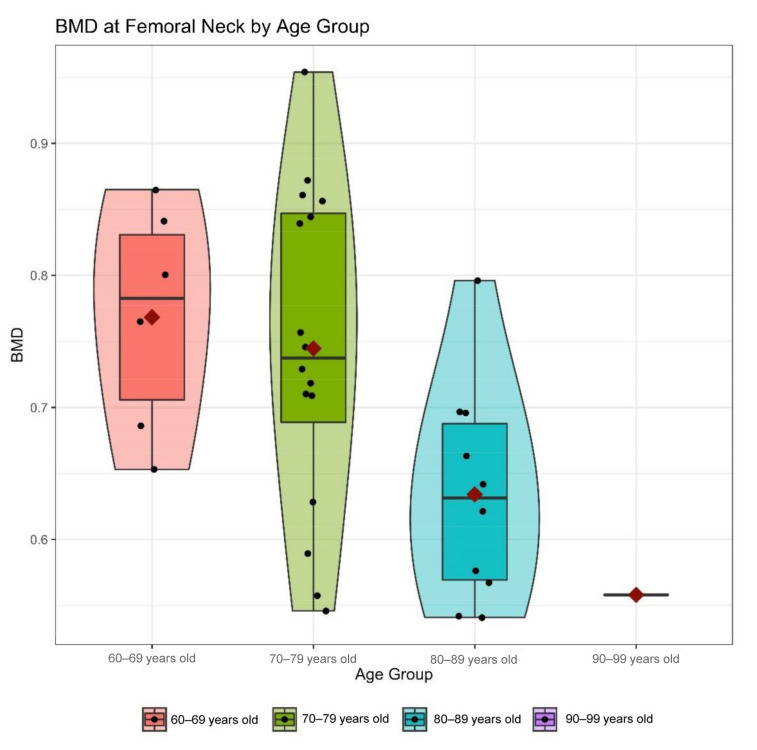
Bone mineral density (BMD) of the femoral neck divided into age groups. There was a continuous decrease of bone density in the collective of distal radius and proximal femur fractures.

In 13 of the 114 patients who had already been diagnosed for osteoporosis before their hospital stay, a revaluation of osteoporosis risk and an adaptation of their drug therapy was performed. The DXA measurement of bone mineral density the bone mineral density (BMD) (Figure 1) at the hip revealed higher T-scores in the group of patients with DRF (−2.13 ± 0.7) compared to the group of patients with proximal femur fractures (−2.74 ± 0.83, *p* = 0.078). The T-score at the lumbar spine showed only a slight difference with −2.38 ± 0.84 in the DRF Group and −2.47 ± 1.27 in the PFF group (*p* = 0.815). A steady decrease in bone density was shown across the age groups (Figure 2).

### 3.3. Patient’s Awareness for Osteoporosis 

**Figure 3:**
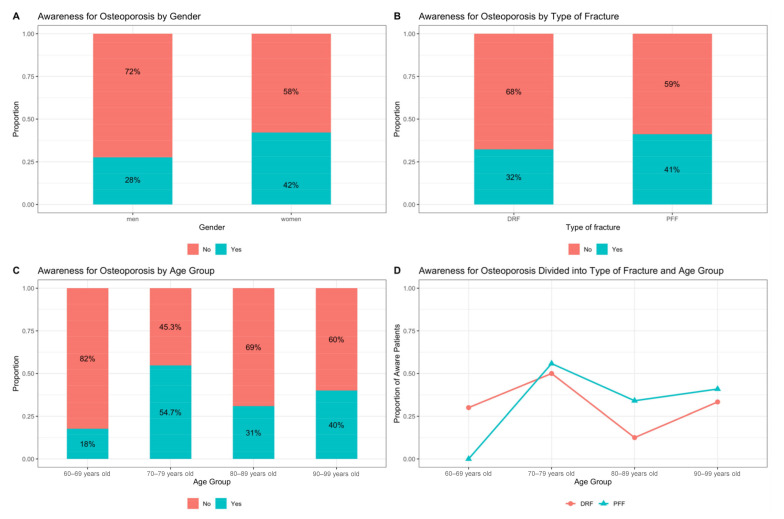
Patients’ awareness: (**A**) Patients’ awareness for osteoporosis divided by gender in percentages; (**B**) Patients’ awareness for osteoporosis divided into distal radius fractures (DRF) and proximal femur fractures (PFF) in percentages; (**C**) Patients’ awareness for osteoporosis divided into fractures (DRF/PFF) and age (four age groups) in percentages; (**D**) Patients’ awareness for osteoporosis divided into fracture types, distal radius fractures (DRF) and proximal femur fractures (PFF) and age (four age groups) in percentages.

According to the questionnaire, awareness was revealed to be higher in the group of patients with proximal femur fractures (41%) compared to the group of patients with distal radius fractures (32%) (Figure 3B). With regards to the gender of patients, it was found that awareness was higher among women (42%) than among men (28%) (*p* = 0.219; two-sample test for equality of proportions with continuity correction) (Figure 3A). Regarding awareness of osteoporosis (AO) in different age groups, it was found that AO was the highest in 70–79-year-olds with 54.7%, and 60–69-year-olds had a significantly lower percentage of awareness with 13% (*p* = 0.017; two-sample test for equality of proportions with continuity correction) (Figure 3C).

If the patients were divided into age groups and into the two types of fracture, it could be seen that in the 60–69 age group, the patients with DRF showed higher awareness in percentage terms at 30% (*n* = 10) than in the PFF group at 0% (*n* = 7). Overall, awareness was the lowest in this group with 18% (*n* = 17). In the three remaining age groups, DRF patients showed lower awareness than the PFF group. For those aged 70–79, awareness was 50% for the DRF group (*n* = 10) and 56% for the PFF group (*n* = 43). For the 80–89 age group, 12% showed awareness in the DRF (*n* = 8) and 34% in the PFF group (*n* = 47). Among those over 90 years of age, the proportion of DRF patients with awareness was 33% (*n* = 3), and the proportion of PFF patients with awareness was 41% (*n* = 22). (Figure 3D).

## 4. Discussion

In elderly trauma patients, the awareness for underlying osteoporosis has a relevant impact for the attendance in further diagnostics and treatment thereof [15]. While therapy adherence of oral bisphosphonates is only 50% after one year, it is obvious that the well-known fracture risk reduction of up to 70% cannot be achieved in dropout patients [16,17]. We found the patients’ willingness to participate in other screening programs (mammography and colonoscopy) to be higher than for osteoporosis, despite already presenting with a fragility fracture and a thereby known risk of further fractures. 

It was shown in the present study that BMD in proximal femur fracture patients was significantly reduced compared to BMD of DRF patients, while there were various risk factors for underlying osteoporosis in all patients. Likewise, the decrease of BMD with age could be shown, which requires therapy adherence, especially in the elderly. As DRFs frequently occur much earlier in life, compared to proximal femur fractures, it appears reasonable that BMD further declines throughout aging, which strengthens the specific context of DRF as an index fracture of underlying osteoporosis. Thus, the necessity arises to connect exactly this patient collective to adequate therapy in the long term. Gong et al. see a gap in care for such patients which should be closed [18]. A gap in care is also emerging in patients with proximal humerus fractures, as this is not classically perceived as an osteoporotic fracture [19].

As shown in the present findings, the awareness of existing osteoporosis was significantly lower in the DRF group than in the PFF group. The underlying causes may be that patients with DRF are hospitalized for significantly fewer days than the PFF subjects [20,21]. This may lead to a lack of time from the physician’s side for detailed osteoporosis education, which may not create sufficient understanding of the severity of the disease. Ashe et al. were able to show that with an appropriate connection, the completion of diagnostics and therapy induction for osteoporosis is possible [22].

Additionally, the impairments in daily activities are substantially lesser in the case of DRF than in the case of a hip fracture. 

Consequently, DRF is not taken as seriously by patients compared to a PFF, and the severity of underlying osteoporosis is likewise underestimated. Due to this misconception, it is understandable that DRF patients are less willing to undergo a more in-depth examination of osteoporosis. Often, the order of causality is not clear to patients that decreased bone stability, rather than the fall itself, is the primary cause of the injury. By analogy, in internal medicine, it is much more common after coronary stent placement to treat the conditions leading to narrowing and calcification of the arteries, such as hypertension and hypercholesterolemia, with long-term medication and regular monitoring. In addition, various structures in the healthcare system often make it difficult to provide consistent therapy. To facilitate this, models such as the Fracture Liaison Service have been established to accompany patients in the long term, even through different sectors of the health system [23,24,25] implementation of an integrated care programme.

Some study limitations have to be considered. The study was carried out in only one university hospital. It would be very interesting to carry it out as a multicenter study. Furthermore, only a limited amount of DRF patients was investigated, which was due to the generally shorter stay in hospital compared to hip fracture patients. Although, patients with cognitive disorders were excluded from the study, it is possible that the individual level of education might affect the patients’ understanding of underlying osteoporosis, while the present study refrained from measuring the educational level.

To the best of our knowledge, the present study was, however, the first study in which the patient’s awareness for underlying osteoporosis when sustaining two frequent fracture entities has been assessed. As DRF remains the most common upper extremity fracture with increasing incidence, more attention should be paid on the identification of underlying osteoporosis, including the patient’s motivation hereof. The present study findings indicate that the patients’ willingness for further osteoporosis diagnostics following DRF was low, which merits more studies into this area. We were able to show that in all groups, the willingness to participate in other screening programs (mammography and coloscopy) was relatively high, significantly higher than willingness to participate in further diagnostics for osteoporosis. Therefore, the implementation of a screening program for osteoporosis such as Fracture Liaison Services (FLS) might improve the patient’s awareness to this disease.

## Figures and Tables

**Table 1:** Demographic data and Characteristics of the study population.

Results	
Age (in years; (%))	79.9 (8.6)
G1 (60–70 years)	20 (13%)
G2 (71–79 years)	54 (36%)
G3 (80–90 years)	62 (41%)
G4 (91–100 years)	14 (9%)
BMI (kg/m^2^)	24.2 (±4.3)
<20 kg/m^2^	12 (8%)
20–24.9 kg/m^2^	80 (53%)
30–34.9 kg/m^2^	41 (27%)
35–39.9 kg/m^2^	14 (9%)
≥40 kg/m^2^	3 (2%)
ASA score	3 (±0.6)
1	7 (5%)
2	59 (36%)
3	80 (53%)
4	4 (3%)
Risk Factors (DVO)	
Parental hip fracture	48 (32%)
Current smoker	13 (9%)
Glucocorticoid use	18 12%)
Rheumatoid arthritis	8 (5%)
Menopause <45 years	46 (38%)
Aromatase inhibitor use	10 (8%)
Alcohol (>1 bottle of beer a day)	19 (13%)
Regular milk product consumption	107 (71%)
Regular vitamin D intake	34 (23%)
Known osteoporosis diagnosis	36 (24%)
Osteoporosis medication intake	23 (68%)

Body mass index (BMI); Physical status classification system (ASA); Dachverband fuer Osteologie (DVO).

## Data Availability

The data presented in this study are available on request from the corresponding author. The data are not publicly available due to privacy and data protection regulations.

## References

[1] Gibson-Smith D., Klop C., Elders P.J.M., Welsing P.M.J., van Schoor N., Leufkens H.G.M., Harvey N.C., van Staa T.P., de Vries F. (2014). The risk of major and any (non-hip) fragility fracture after hip fracture in the United Kingdom: 2000–2010. Osteoporos. Int..

[2] Sakuma M., Endo N., Oinuma T., Endo E., Yazawa T., Watanabe K., Watanabe S. (2008). Incidence and outcome of osteoporotic fractures in 2004 in Sado City, Niigata Prefecture, Japan. J. Bone Miner. Metab..

[3] Jupiter J.B. (1991). Current concepts review. Fractures of the distal end of the radius. J. Bone Jt. Surg. Ser. A.

[4] Jerrhag D., Englund M., Karlsson M.K., Rosengren B.E. (2017). Epidemiology and time trends of distal forearm fractures in adults—a study of 11.2 million person-years in Sweden. BMC Musculoskelet. Disord..

[5] Cuddihy M.T., Gabriel S.E., Crowson C.S., O’Fallon W.M., Melton L.J. (1999). Forearm fractures as predictors of subsequent osteoporotic fractures. Osteoporos. Int..

[6] Kanis J.A., Odén A., McCloskey E.V., Johansson H., Wahl D.A., Cooper C. (2012). A systematic review of hip fracture incidence and probability of fracture worldwide. Osteoporos. Int..

[7] Downey C., Kelly M., Quinlan J.F. (2019). Changing trends in the mortality rate at 1-year post hip fracture—a systematic review. World J. Orthop..

[8] Dyer S.M., Crotty M., Fairhall N., Magaziner J., Beaupre L.A., Cameron I.D., Sherrington C. (2016). A critical review of the long-term disability outcomes following hip fracture. BMC Geriatr..

[9] Desai R.J., Mahesri M., Abdia Y., Barberio J., Tong A., Zhang D., Mavros P., Kim S.C., Franklin J.M. (2018). Association of Osteoporosis Medication Use After Hip Fracture With Prevention of Subsequent Nonvertebral Fractures: An Instrumental Variable Analysis. JAMA Netw. Open.

[10] Saklad M. (1941). Grading of Patients for Surgical Procedures. J. Am. Soc. Anesthesiol..

[11] Doyle D.J., Goyal A., Bansal P., Garmon E.H. (2020). American Society of Anesthesiologists Classification.

[12] Pfeilschifter J. (2015). Osteoporose-Diagnostik: Was ist neu in der DVO-Leitlinie 2014?. Dtsch. Med. Wochenschr..

[13] Neuerburg C., Mittlmeier L., Schmidmaier R., Kammerlander C., Böcker W., Mutschler W., Stumpf U. (2017). Investigation and management of osteoporosis in aged trauma patients: A treatment algorithm adapted to the German guidelines for osteoporosis. J. Orthop. Surg. Res..

[14] Cosman F., de Beur S.J., LeBoff M.S., Lewiecki E.M., Tanner B., Randall S., Lindsay R. (2014). Clinician’s Guide to Prevention and Treatment of Osteoporosis. Osteoporos. Int..

[15] Boudreau D.M., Yu O., Balasubramanian A., Wirtz H., Grauer A., Crittenden D.B., Scholes D. (2017). A Survey of Women’s Awareness of and Reasons for Lack of Postfracture Osteoporotic Care. J. Am. Geriatr. Soc..

[16] Ziller V., Kostev K., Kyvernitakis I., Boeckhoff J., Hadji P. (2012). Persistence and compliance of medications used in the treatment of osteoporosis—Analysis using a large scale, representative, longitudinal German database. Int. J. Clin. Pharmacol. Ther..

[17] Murad M.H., Drake M.T., Mullan R.J., Mauck K.F., Stuart L.M., Lane M.A., Abu Elnour N.O., Erwin P.J., Hazem A., Puhan M.A. (2012). Comparative effectiveness of drug treatments to prevent fragility fractures: A systematic review and network meta-analysis. J. Clin. Endocrinol. Metab..

[18] Hyun S.G., Won S.O., Moon S.C., Joo H.O., Young H.L., Goo H.B. (2009). Patients with wrist fractures are less likely to be evaluated and managed for osteoporosis. J. Bone Jt. Surg. Ser. A.

[19] Kim T.I., Choi J.H., Kim S.H., Oh J.H. (2016). The adequacy of diagnosis and treatment for osteoporosis in patients with proximal humeral fractures. CiOS Clin. Orthop. Surg..

[20] Dimai H.P., Svedbom A., Fahrleitner-Pammer A., Resch H., Muschitz C., Thaler H., Szivak M., Amrein K., Borgström F. (2014). Epidemiology of distal forearm fractures in Austria between 1989 and 2010. Osteoporos. Int..

[21] Lippuner K., Von Overbeck J., Perrelet R., Bosshard H., Jaeger P. (1997). Incidence and direct medical costs of hospitalizations due to osteoporotic fractures in Switzerland. Osteoporos. Int..

[22] Ashe M., Khan K., Guy P., Kruse K., Hughes K., O’Brien P., Janssen P., McKay H. (2004). Wristwatch—Distal radial fracture as a marker for osteoporosis investigation: A controlled trial of patient education and a physician alerting system. J. Hand Ther..

[23] Åkesson K.E., McGuigan F.E.A. (2021). Closing the Osteoporosis Care Gap. Curr. Osteoporos. Rep..

[24] Gosch M., Kammerlander C., Neuerburg C. (2019). Osteoporosis—Epidemiology and quality of care. Z. Gerontol. Geriatr..

[25] Geiger I., Kammerlander C., Höfer C., Volland R., Trinemeier J., Henschelchen M., Friess T., Böcker W., Sundmacher L., FLS-CARE study group (2021). Implementation of an integrated care programme to avoid fragility fractures of the hip in older adults in 18 Bavarian hospitals—study protocol for the cluster-randomised controlled fracture liaison service FLS-CARE. BMC Geriatr..

